# Supplementary material for: QTL Mapping of Yield, Agronomic, and Nitrogen-Related Traits in Barley (Hordeum vulgare L.) under Low Nitrogen and Normal Nitrogen Treatments
Source: Plants (Basel). 2024 Aug 1;13(15):2137. doi: 10.3390/plants13152137 (PMC11314459; doi:10.3390/plants13152137)
Supplement: Supplementary file 1 [file plants-13-02137-s001.zip › plants-3065644-supplementary.pdf]

Table S1. QTL cluster for more than three QTL

Cluster codes	Chr	Marker interval	No. Of QTLs	QTLs' name
C1	5H	*bpb5179-bpb1494*	3	*Qldr.sau-5H.1, Qgp.sau-5H.1, Qnutedm.sau-5H*
C2	5H	*bpb8462-bpb3241*	4	*Qhi.sau-5H, Qldr.sau-5H.2, Qstna.sau-5H, Qnhi.sau-5H*

Table S2. summary of candidate genes

QTLs	Putative candidate gene ID	Gene annotation
Qtgw.sau-2H	*HORVU.MOREX.r3.2HG0169570.1*	NAC domain-containing protein
	HORVU.MOREX.r3.2HG0171720.1	Glutamate decarboxylase
	HORVU.MOREX.r3.2HG0177640.1	B-box zinc finger protein
	HORVU.MOREX.r3.2HG0184930.1	Zinc finger, B-box
	HORVU.MOREX.r3.2HG0189840.1	Isoaspartyl peptidase/L-asparaginase
	HORVU.MOREX.r3.2HG0202350.1	Glutamine synthetase
Qph.sau-3H, Qldr.sau-3H	*HORVU.MOREX.r3.3HG0229730.1*	Isoaspartyl peptidase/L-asparaginase
	HORVU.MOREX.r3.3HG0231360.1	NAC domain-containing protein
	HORVU.MOREX.r3.3HG0232400.1	Glutamate decarboxylase
	HORVU.MOREX.r3.3HG0236050.1	Basic helix loop helix (BHLH) family transcription factor
	HORVU.MOREX.r3.3HG0243610.1	B-box zinc finger family protein
	HORVU.MOREX.r3.3HG0249810.1	Basic helix-loop-helix (BHLH) family transcription factor
	HORVU.MOREX.r3.3HG0287730.1	Glutamate carboxypeptidase 2
	HORVU.MOREX.r3.3HG0296060.1	NAC domain protein
Qldr.sau-5H.1,Qgp.sau-5H.1	*HORVU.MOREX.r3.5HG0504130.1*	NAC domain protein
	HORVU.MOREX.r3.5HG0504140.1	NAC domain protein
	HORVU.MOREX.r3.5HG0509430.1	Basic helix-loop-helix (BHLH) family transcription factor
	HORVU.MOREX.r3.5HG0517260.1	Glutamate carboxypeptidase 2
QnuteGY.sau-7H	*HORVU.MOREX.r3.7HG0727850.1*	Basic helix loop helix (BHLH) family transcription factor
	HORVU.MOREX.r3.7HG0729020.1	High-affinity nitrate transporter 2.2
